# An Account of the North Kensington Friendly Workers among the Poor

**Published:** 1892-10-01

**Authors:** C. Furley Smith


### THE CURE OF POVERTY,

### AN ACCOUNT OF THE NORTH KENSINGTON FRIENDLY WORKERS AMONG THE POOR —(*concluded from page* 432, *Vol. XII.*).

By Mrs. C. Furley Smith.

This seems incredible, and can only be excused on the ground that the inhabitants of North Kensington do not appreciate properly the work done for them by the Friendly Workers. They do not realise that this Association has successfully administered the charities of the district. That, by bringing into harmonious co-operation all the ministers of religion and trustees of charitable funds in the district, they have been able to bring every poor person under the notice of those most interested in helping him; and at the same time have prevented that duplication of charities by which the unworthy profit and the worthy are cheated. That being thus in the confidence of the charitable of the district, they have been able to give letters of admission to hospitals and convalescent homes, surgical instruments and other aids when wanted, besides clothing, coals, and other gifts, and to distribute them to the best advantage. That having earned the respect of employers of labour throughout the district, they have been able to find work for many people who might otherwise have become paupers, and degenerated into being a permanent burden on the rates. That having won the confidence of the poor, they have been called in to advise and help in cases of sanitary defect, which might have proved centres of infectious disease, that would have become epidemic in the district; and in cases where disreputable inhabitants had taken up their abode, thereby tending to destroy the respectability of the district, and to lower the value of property. Besides this, children have been removed from bad surroundings; foster-mothers (not baby-farmers) have been found for orphans; nurses have been sent to cases of sickness; the old and infirm have been tended in their last days; neglectful husbands have been compelled to maintain their wives and children; the innumerable emergencies arising from sickness in families have been dealt with and tided over; tools and clothing have been redeemed from pawn, and the industrious and unfortunate have been made self-supporting once more. Every form of poverty and misery that has appeared has been dealt with successfully—there has never been a question of the success. And the secret of the success has been this—personal knowledge of the poor, personal devotion to them. Yet the people of North Kensington will not provide the means for a man to acquire this knowledge and to give this devotion.

It is true that in the balance-sheet there does appear this sum of £99 for salaries. But what does this mean? The fact is that for eighteen months Mr. Mackenzie gave his services gratuitously, and that from the beginning of 1891 he has received a salary, not from the funds of the Association, but from two gentlemen, Mr. Henry C. Burdett and Mr. Leopold de Rothschild. As far as the mass of the population of North Kensington is concerned, they have not paid one penny. It seems then that they are willing to give the sum (about £100 a-year) which is necessary for the administration of charity and supply of immediate relief in their district; but that they will not give another £100 to pay the salary of a secretary to investigate cases and administer relief. Without such a secretary the Association cannot go on. In addition to the desultory help of other charitable workers, of which we would speak with the utmost gratitude and appreciation, it is necessary that there should be one man always ready to do the work for which the Association was formed, a man who shall make the needs of the North Kensington poor the chief business of his life. Such men can be found, men who are willing to give themselves to this work; but of such, how many have private means sufficient to render them independent of a salary? And if such came in thousands, is it just that they should be asked, or even permitted, to do, as a charity to the well-to-do inhabitants of North Kensington what is admittedly a work of public benefit? What if there does come a candidate who can afford to do without the £100 a year! That would not justify North Kensington in taking as a charity what ought to be paid for as a matter of business. Would anyone cut off the Prime Minister's official income on the plea that he can do without it? His private means, be they large or small, do not affect the public duty of payment for work done for the public good. North Kensington can, if it will, raise the salary for Mr. Mackenzie's successor; North Kensington is in duty bound to do so. To take a secretary's laborious and devoted work as a gift either from himself or through the generosity of Mr. Leopold de Rothschild and Mr. Burdett is to become themselves recipients of charity on a tolerably large scale. Not that £100 is adequate payment for Mr. Mackenzie or for a successor like him. Do not let us imagine that. All that can be said of it is that it will make it possible for a man whose heart is already in such work to follow it systematically, without the distraction of having to turn to other employment in order to obtain the necessaries of life.

Imagination would like to picture a North Kensington whose inhabitants would say, "This is our own scheme for relieving the necessities of the poor. It is simple, it is economical, it has been most successful. On winter nights we can lie down in our beds feeling sure that no worthy inhabitant of our district stands in need of warmth and food and cannot obtain it. On Sundays we can go to church and bow in prayer and lift up our voice in praise, sure that while we share in the privilege of worship, and fulfil the minor duty of meeting together in God's name and in His house, we are not forgetting the greater matters of the law; sure that we are doing our best to realise the close connection between the two commands, 'Thou shalt love thy God with all thy heart' and 'Thou shalt love thy neighbour as thyself.' We will not let this plan fall to the ground. Rather will we strive to make its workings so successful and so conspicuous in their success that every district in London shall imitate us, and have Friendly Workers of its own."

What a London that would be—and easily might be—where every poor man had a friend, every widow an adviser, every orphan a helper and a guide. Where the industrious were helped to work, and the sick brought back to health; where all our great and costly agencies of help, now working blindly and in ignorance of each other's efforts, were systematised and shown the paths of fullest usefulness.

The swindler, the professional beggar, the whining supplicant at every open door, would be found out, and treated according to his deserts; the worthy poor would be helped in the way each most needed to take him out of hopelessness and hunger; workers among the poor would come to know each other, and help each other by each bringing before the other the cases each could most adequately aid. So much has been done in one district; so marvellously much might be done in all; and North Kensington might show the way.

"But for the sake of an extra hundred pounds a year to pay the man who shall be the ready hands, the willing feet, the loving heart, the thoughtful brain of the whole Association, it gave up the honour, the privilege of guiding the whole great city."

*North Kensington, shall this be said of you?*

